# A mixed methods study investigating patient's initial experiences after acquiring a surgical scar, burn injury, and/or amputation

**DOI:** 10.1177/20595131251387481

**Published:** 2025-10-27

**Authors:** Philippa Tollow, Georgia Mouhlen, Maia Thornton

**Affiliations:** Centre for Appearance Research, University of the West of England, Bristol, UK

**Keywords:** Appearance, surgical scar, burn injury, amputation, distress, preparedness, support

## Abstract

**Introduction:**

Experiences of seeing an altered appearance for the first time can have an important influence on an individual's psychological wellbeing, their body image and their adjustment. Existing research has explored this experience in individuals with breast cancer and facial burns, but limited research has been conducted in other appearance altering conditions. The aim of this study was to explore patient's initial experiences viewing themselves after acquiring an appearance altering condition, including distress, preparedness and social support.

**Methods:**

Participants (*n* *=* 64) with a surgical scar, burn injury or amputation completed a mixed methods online survey. This survey included questions about practical elements of the experience, the support they received, their expectations, distress, and preparedness; as well as open questions regarding participant's feelings, fears, support and suggested improvements to the experience. Data analysis included statistical analysis and inductive content analysis.

**Discussion:**

Participants reported a variety of initial experiences. Some felt adequately prepared and supported, whilst others felt this could have improved. A strong correlation was found between perceived support and distress (r_s_ = −.66, *p* = <.001) and preparedness and distress (r_s_ = −.66, *p* = <.001). No significant differences in preparedness, distress or support were found according to gender, age, or condition.

**Conclusions:**

Whilst many participants reported satisfaction with viewing their appearance for the first time, some participants reported significant distress and a need for further support around this experience. This suggests an important role for healthcare providers in discussing expectations and providing appropriate patient support following injury or treatment.

## Plain language title

Patients experiences of seeing their altered appearance for the first time after surgery, a burn injury, or amputation.

**Plain language summary:**

**Purpose of the Study**

This study aimed to understand how people feel when they first see their appearance after experiencing a change due to a surgical scar, burn injury, or amputation. The focus was on their distress, how prepared they felt, and the social support they received.

**How the Study Was Done**

A total of 64 participants with a surgical scar, burn injury, or amputation took part in an online survey that included both closed-ended and open-ended questions. The researchers analysed the responses using statistical methods, as well as looking for common themes in the open-ended answers.

**What the Study Found**

Participants had a range of initial reactions, with many experiencing some level of distress. Some felt well-prepared and supported, while others thought there was room for improvement. The study found that feeling prepared was strongly linked to having good social support.

**Conclusions**

Although many participants were happy with the care they received, some experienced significant distress and felt they needed more support when first seeing their changed appearance. This highlights the importance of healthcare providers discussing what to expect and offering appropriate support to patients after an injury or treatment.

## Introduction

A visible difference is defined as an appearance that is different to culturally defined norms. This may be congenital (e.g., cleft lip and palate) or acquired through treatment or accident (e.g., burns).^1^ Whilst individual experiences differ, we know that many people with appearance altering conditions experience negative reactions from others, including staring and unwanted comments, as well as experiencing higher levels of appearance-related distress, self-esteem issues, anxiety and depression.^2^ Previous research has suggested that an altered appearance after surgery can have a severe and long-lasting impact on body image and appearance related distress,^3,4^ with some indication that lower levels of social support may be a predictor of some of the negative outcomes experienced.^5^ However, whilst much research has focused on the longer-term psychosocial outcomes of individuals with a visible difference, there is little research looking into individual's initial experiences surrounding acquiring an appearance altering condition, including seeing their appearance change for the first time.

One factor that may have an important influence on patients’ perception of their visible difference could be patients’ initial experiences of seeing their appearance. In one study exploring patient' post-burn surgery experiences, it was noted that a critical incident for future body image dissatisfaction was looking in the mirror for the first time after surgery following a burn injury. Participants reported this event being associated with fear and shame surrounding their body image and, in some cases, led to individuals displaying avoidance behaviour, such as not following care procedures.^6^ Further research has been conducted exploring initial experiences after acquiring a facial burn, finding a significant negative association between perceived social support and levels of distress,^5^ with similar findings reported in women who have had mastectomy and/or breast reconstruction surgery.^7^ The latter study conducted an exploratory mixed methods survey looking into women's experiences of breast reconstruction surgery, finding that 71% of their sample reported feeling worried about looking at their change in appearance for first time. Thematic analysis of the same dataset found that “preparedness and support” was a key theme, with participants reporting a variety of experiences in terms of their preparedness to see their appearance.^8^ These studies suggest that viewing an altered appearance for the first time is an important time point for individuals who had experienced a change to their appearance, whether their experience was positive or negative, and that both social support and preparedness played a key role in determining this.

These findings are also supported by psychological theory emphasising the importance of this timepoint in a patient's recovery. For example, Cash's Cognitive-Behavioural Model of Body Image suggests that critical events, such as a change in appearance, can activate scheme-driven processing of information related to one's appearance. These will influence internal dialogues, such as automatic thoughts, social comparisons and interpretations of the altered appearance, and potentially have a significant impact on the individual's emotional state. Thus, the ‘first look’ at their altered appearance may set the tone for subsequent coping.^9^ Similarly, this experience could be viewed through the lens of Lazarus and Folkman's Cognitive Appraisal Theory,^10^ with the first viewing of an altered appearance representing a ‘primary appraisal moment’, where the individual is confronted with the question of what this means for them. Their secondary appraisal of the situation then involves assessing whether they have the coping skills or resources available to manage this situation and their reaction, with subsequent stages of stress, coping skills and reappraisal. Both theories, supported by the existing evidence in this area, underpin the importance of this experience in adjustment to an altered appearance.

Whilst individuals’ experience of viewing their appearance for the first time is a growing and important area of research, samples have previously been limited to individuals who have had surgery for breast cancer or who have experienced a facial burn. It is not clear to what extent these experiences are shared with individuals who have other acquired appearance-altering conditions. Whilst the experience across conditions involves elements of expectations, preparation and appearance beliefs, there may also be differences according to whether this is the result of an injury or illness, whether this change in appearance was anticipated, and the team involved in their care. Similarly, previous studies in this area have not found evidence that demographic factors, such as age or gender, may influence this experience, with women who have experienced a mastectomy reporting no significant differences according to age, marital status or type of procedure in terms of concern at seeing their breast for the first time, feeling ready or finding the experience distressing.^7^ Likewise, no demographic differences according to age, cause of burn, total burn surface area, or inpatient admission was found when exploring the amount of distress when looking at the appearance of a facial burn for the first time.^5^

It is not clear whether these demographic differences will also be absent in other conditions. Therefore, this study aimed to expand upon previous research and explore initial experiences of individuals with a range of appearance altering conditions, including further exploration of the relationship between preparedness and distress, and the influence of demographic factors. Specifically, this study aims to answer the following research questions:

1. What are the initial experiences of individuals who are seeing their appearance for the first time after appearance-altering surgery, amputation or burn injury?
2. Is there an association between preparedness and distress when viewing an appearance for the first time after acquiring a change to appearance?
3. Is there an association between demographic factors (age, gender, cause of appearance change) and distress, preparedness or support when viewing their appearance for the first time?

## Methods

### Design and measures

This study followed a questionnaire design, using an online survey to collect quantitative and qualitative data regarding individual's initial experiences of viewing their appearance after acquiring a visible difference. The contents of this survey were adapted from existing research investigating patients’ initial experiences of viewing their appearance after sustaining a facial burn injury.^5^ Ethical approval for this research was sought from the ‘University of the West of England Psychology Ethics Committee’ (ref number: VCMSC20212211), with consideration of factors such as confidentiality, data security and participant welfare. For example, participants were reassured that all their data would be kept confidential, with any potentially identifiable information removed or anonymised when compiled for analysis. Only the researchers conducting this study would have access to the data, which was stored in accordance with the Data Protection Act 2018 and General Data Protection Regulation requirements. Before taking part in the research participants were made aware of the topics that would be included and the potential for some participants to find these distressing. Participants were encouraged to consider this before taking part and everyone considering participation received information of local and national support resources that were available to them regardless of whether or not they chose to take part. All participants were required to read a Participant Information Sheet and provide written consent before taking part, including written consent to publish.

After providing consent to take part in the study, participants were asked to complete demographic questions and questions relating to their altered appearance, including when their appearance changed, where on their body they experienced this change, and whether it was expected (e.g., a planned surgery) or unexpected (e.g., an injury event). This was followed by six questions about participants thoughts, behaviours, and expectations before seeing their altered appearance for the first time, one example states: “Before I saw my altered appearance, I was worried about seeing it for the first time.” All questions were measured on a Likert scale ranging from 1 (strongly agree) to 5 (strongly disagree). A further three questions asked the participants where they were when they first saw their altered appearance, who was with them, and whose idea it was to look at their altered appearance for the first time. Four more questions asked about the participants’ distress, expectations, and preparedness surrounding seeing their appearance altering condition for the first time. Two of these questions were on a 1–10 Likert Scale (e.g., 1 = not at all distressed/not at all prepared, 10 = extremely distressed/completely prepared), whilst the other two were multiple choice questions asking about the images participants saw beforehand and their expectations. Participants were then asked three questions about the support they received around seeing their appearance altering condition for the first time on a 1 (strongly agree) to 5 (strongly disagree) Likert Scale. Finally, four open-ended questions were asked to gain a deeper understanding of participants feelings, fears, support, and improvements surrounding their initial experiences seeing their altered appearance for the first time. All questions were adapted from existing research investigating patients’ initial experiences of viewing their appearance after sustaining a facial burn injury,^5^ including replication of the same Likert skills and response categories. This is the same methodology that has been adapted by other studies on this topic, with the aim of increasing the comparability of results across studies.

### Participants

Participants were eligible to take part in this study if they had experienced a new surgical scar, burn injury and/or amputation at least six months previously. A minimum time of six-months post-appearance change was chosen to reduce the likelihood of experiencing significant distress through recounting their initial experiences and to provide time for reflection since the experience. Participants were also required to have sufficient understanding of written English to complete a survey and provide informed consent (resources were unfortunately not available for translations). Participants were recruited via social media and via email to a participant pool held by the researcher's institution, which included individuals wishing to take part in appearance related research. The recruitment advert outlined the purpose of the study, indicated that it was a mixed methods survey, and gave the approximate time to complete. No monetary incentive was offered for completion. A G*Power calculation for correlation analysis was performed a priori to determine the number of participants required, this was based on a 0.3 effect size, a significance level of 0.05, and a 95% confidence interval. This suggested 134 participants would be required. Unfortunately this sample was not achieved, however, all statistical analyses performed did meet the necessary assumptions. This is further reflected upon in the ‘Limitations’ section.

A total of 82 participants responded to an advert to complete an online survey of participant' initial experiences of an acquired visible difference. Of these 82 participants, 66 were deemed eligible after self-identifying that they had experienced either a new surgical scar, burn injury and/or amputation at least six-months ago. Those who did not have one of these appearance altering conditions (*n* = 5); or had the appearance altering condition for less than six months (*n* = 11) were directed towards further support and not invited to take part in the study. A further two participants were excluded as they reported not remembering their initial experiences of seeing their appearance altering condition. Therefore, a total of 64 participants were included in this study. Participants were aged 18–62 years old (M = 39.00 years; SD = 13.87), and all had either a burn injury, surgical scar, and/or amputation. Participants were predominantly female (76.2%) and of White ethnicity (88.9%). Unfortunately, data was not collected on the reason for the surgical scar, and it should be noted that only one participant had experienced an amputation. See Table 1 for a detailed overview of participant demographics.

**Table 1.** Participant demographics.

Characteristics		N	%
Gender	Male	13	20.3
	Female	49	76.6
	Non-Binary	2	3.1
Age range	18–24	13	20.3
	25–54	34	53.1
	55–74	16	25.0
	75+	1	1.6
Time since appearance altering condition	6–9 months	5	7.8
	9–12 months	2	3.1
	12–18 months	3	4.7
	18 months +	54	84.4
Appearance altering condition	Surgical scar	50	78.1
	Burn injury	13	20.3
	Surgical scar and amputation	1	1.6
Ethnicity (*n* *=* 64)	Asian/British Asian	2	3.1
	Mixed Background	2	3.1
	White	57	89.1
	Other	1	1.6
	Prefer not to say	2	3.1

### Analysis

Statistical analysis was conducted using SPSS. Firstly, descriptive statistics and frequencies were explored, followed by normality tests. The data demonstrated a non-normal distribution, thus non-parametric correlation analyses were used to analyse the relationships between the quantitative variables. Mann Whitney U or Kruskal-Wallis tests were used to analyse the differences between variables.

Qualitative data was analysed using a qualitative content analysis, this was an inductive content analysis as no themes were preconceived prior to analysing the data. Categories and sub-categories were identified, and the frequency of occurrence was then calculated. The steps followed were open coding, creating categories, and abstraction.^11^ Analysis was conducted by the second author, with supervision from the first and third authors.

## Results

### Prior experiences and worst fears

As Table 2 reports, 69% (n = 44) of participants were worried about seeing their appearance for the first time, and 67% (n = 43) were concerned about what their appearance would look like. Seventy-eight per cent felt it was important to look at their altered appearance. Thirty-one per cent initially avoided looking at their altered appearance for the first time, and 52% reported having negative images in their mind before seeing their appearance for the first time. Thirty-seven per cent tried to ‘read’ other people's reactions to gauge what they looked like.

**Table 2.** Patient's experiences prior to seeing their altered appearance for the first time.

	Strongly agree	Somewhat agree	Neither agree nor disagree	Somewhat disagree	Strongly disagree
Before I saw my altered appearance, I was worried about seeing it for the first time.	16 (25%)	**28** **(** **43.8%)**	3 (4.7%)	9 (14.1%)	8 (12.5%)
Before I saw my altered appearance, I was concerned about what I would look like.	17 (26.6%)	**26** **(** **40.6%)**	4 (6.3%)	9 (14.1%)	8 (12.5%)
I felt it was important for me to look at my altered appearance.	**26** **(** **40.6%)**	24 (37.5%)	8 (12.5%)	2 (3.1%)	4 (6.3%)
I initially avoided or put off looking at my altered appearance for the first time.	14 (21.9%)	6 (9.4%)	5 (7.8%)	**20** **(** **31.3%)**	19 (29.7%)
Before I looked at my altered appearance, I had negative or unpleasant images in my mind about what I might look like.	12 (19%)	**21** **(** **33.3%)**	12 (19.0%)	8 (12.7%)	10 (15.9%)
Before I looked at my altered appearance, I tried to ‘read’ other people's reactions to me as a way of gauging what I looked like.	7 (11.1%)	16 (25.4%)	10 (15.9%)	9 (14.3%)	**21** **(** **33.3%)**

*Note*. Bolded values signify the most common response.

Sixty-two participants (97%) answered an open-ended question about their worst fears regarding seeing their altered appearance for the first time, including any images they had in mind prior to seeing their appearance change. Content analysis was used to sort these responses into four categories: “Neutral” (n = 13; e.g., “I wasn’t quite sure what to expect”), “Social” (n = 13; e.g., “People are going to judge me and think I’m ugly”), “Appearance” (n = 40; e.g., “Worried about how it would look, would it be grotesque?”) and “Medical” (n = 3; e.g., “Infection fear”). The sub-categories and examples drawn from the data are displayed in Table 3, demonstrating the worst fears and images participants had in mind prior to seeing their altered appearance for the first time. These support the quantitative findings and suggest that many of the fears that individuals had around this timepoint were related to their appearance, with a focus on uncertainty about how their appearance might have changed, concerns around how extreme the change to their appearance would be and the permanence of this change.

**Table 3.** Content analysis of responses to questions: “What was your worst fear about seeing your altered appearance for the first time? What thoughts or images went through your mind?”

Category (n)	Sub-Category	Frequency	Example
Neutral (13)	No predetermined fears	12	“I had no fears.”
	Not sure what to expect	1	“I wasn't quite sure what to expect…”
Social (13)	Other people's perceptions/reactions	9	“People are going to judge me and think I’m ugly.” “People would treat me differently”
	Negative impact upon relationships	3	“If it would hinder relationships with anyone.”
	Where to get help	1	“Where to go for help!”
Appearance (40)	Concern about how the new appearance will look	27	“Worried about how it would look, would it be grotesque?” “I was concerned how bad the scar would be…”
	Feeling unattractive	6	“I would be unattractive” “In case it looked ‘ugly'”
	Fear of permanence	7	“That it would be permanent” “That I'll have visible scars forever.”
Medical (3)	Fear of infection	3	“Infection fear”

### Distress, expectations, preparedness, and support

In terms of who patients were with when seeing their appearance for the first time, 39% were with friends or family (n = 25), 38% were alone (n = 24), 38% were with a nurse (n = 24), 20% (n = 13) were with a doctor, and 2% of individuals were with an employer (n = 1) or could not remember (n = 1) (*Note: Participants were able to select more than one option if multiple people were present)*. Most participants decided to look at their appearance altering condition for the first time themselves (n = 46; 72%), and eight participants reported that it was a medical professional's idea (13%).

When asked how distressing seeing their appearance had been for the first time on a scale of 0–10 (0 being “not at all distressing”, 10 being “extremely distressing”), the mean response was 4.53 (range = 0–10, SD = 2.92). Thirty eight percent (n = 24) of participants found the experience less distressing than they expected it to be, whereas 20% percent of participants found the experience more distressing than they were expecting it to be (n = 13), the rest of the participants reported that their experience was about the same as they expected it to be (n = 27; 42%). Thirty percent of participants reported that the image they saw was better than the image they had in mind (n = 19); 33% reported that the image they saw is what they expected it to be (n = 21); 16% reported the image to be worse than their expectations (n = 10); and 22% reported having no images in mind beforehand (n = 14). Lastly, the mean preparedness rating on a scale of 0–10 for participants was 5.86, where 0 = “not at all ready”, and 10 = “completely ready”.

Sixty-two participants (97%) answered an open-ended question asking them to describe the feelings and emotion they had when seeing their altered appearance for the first time. Four categories were created based on the data: “Positive” (n = 19; e.g., “I had expected far worse scarring”), “Neutral” (n = 16; e.g., “As expected, just glad it was done”), “Fear” (n = 17; e.g., “I was scared to look to start off with…”), and “Distress” (n = 36; e.g., “I was shocked, it looked horrible”), and the sub-categories, frequencies and examples can be found in Table 4. The large number of individuals whose feelings and emotions around this experience could be categorised under ‘distress’ provides an added level of nuance to the quantitative results and suggests that many of those who did perceive this event as distressing experienced strong emotions to this effect. In addition, it 's notable that almost a third of participants reported positive experiences in their qualitative answers and approximately a quarter reported neutral responses. Both results add further evidence of the wide range of experiences reported by this sample.

**Table 4.** Content analysis of responses to question: “Please describe the feelings or emotions you had when you saw your altered appearance for the first time.”

Category (n)	Sub-Category	Frequency	Example
Positive (19)	Better than expected	7	“I had expected far worse scarring…” “ I didn’t expect it to have healed as well…”
	Relief	5	“Relief that it wasn't as visually bad…”
	Thankful	4	“…bigger picture glad to be alive.”
	Surprised	2	“…I was pleasantly surprised…”
	Reassured	1	“…I felt reassured that it wouldn't look like that forever.”
Neutral (16)	As expected	5	“As expected, just glad it was done”
	Other priorities	4	“…the surgical scars were not the greatest concern as I was close to death for a while.
	Unemotional	3	“No emotions…”
	No memory	3	“…I don't remember much.”
	Curiosity	1	“…my emotion went from fear to interest around how the injury looked…”
Fear (17)	Anxiety	6	“Anxious about how long it would take these surgical scars to heal and fade and to look ‘normal’ ish again” “I was very apprehensive and worried about looking…”
	Scared	5	“I was scared to look to start off with…”
	Permanence	5	“I realised that this would be forever.”
	Vulnerable	1	“I felt mutilated, damaged, and incredibly vulnerable…”
Distress (36)	Shock	8	“I was shocked, it looked horrible.” “…I was shocked, I hadn’t had a vision in mind, it looked a mess…”
	Upset	8	“I was upset” “I felt sad as I thought it looked ugly…”
	Anger	6	“I was angry as it was result of a workplace incident…” “I felt angry at being put in the position of having no choice…”
	Disappointed	4	“It was what I expected to see, but I was still disappointed when I did see it.”
	Pain	3	“I was in a lot of pain”
	Insecure	3	“…feel insecure about the scar appearance…”
	Shame	2	“I was ashamed…”
	Disgust	2	“It filled me with disgust to look at the burn area”

Strong significant associations were found between perceived support and distress (r_s_ = −.66, *p* = <.001), and preparedness and distress (r_s_ = −.66, *p* = <.001). This indicates that participants with higher levels of distress were also more likely to report lower levels of social support and preparedness (see Table 5). This also echoes the qualitative findings, with many of those reporting positive or neutral emotions around this experience making reference to their experience matching their expectations or being better than they expected.

**Table 5.** Correlations between variables.

		Distress	Preparedness	Perceived support
Spearman's rho	Distress	1.000	−.664**	−.666**
	Preparedness	−.664**	1.000	.610**
	Expectations	.274*	−.072	−.028
	Perceived Support	−.651**	.625**	1.000

*p < .005 and ** *p* < .001.

### Perceived support experiences

Participants answered three questions regarding the amount of perceived support they had when seeing their appearance altering condition, using a scale of 1–5 (1 = strongly disagree, 5 = strongly agree). Full details of these results can be found in Table 6. Thirty-two participants (50%) felt they received enough help when seeing their appearance altering condition for the first time, whereas sixteen participants (25%) felt they did not receive enough help.

**Table 6.** Results of participants’ perceived social support.

	Strongly disagree	Somewhat disagree	Neither agree nor disagree	Somewhat agree	Strongly agree
I feel I received enough help when seeing my altered appearance for the first time	5 (7.8%)	11 (17.2%)	16 (25.0%)	15 (23.4%)	**17** **(** **26.6%)**
I would have valued more support around the area of seeing my altered appearance for the first time	7 (10.9%)	**18** **(** **28.1%)**	**18** **(** **28.1%)**	12 (18.8%)	9 (14.1%)
I believe my experience in the amount of care received could have been improved	9 (14.1%)	**16** **(** **25.0%)**	**16** **(** **25.0%)**	13 (20.0%)	10 (14.1%)

Sixty-two participants (97%) answered two additional open-ended questions about the help that was offered (see Table 7) and whether this could have been improved (see Table 8) when seeing their appearance altering condition for the first time. Whilst 32 participants did not have any suggestions for improvements to support, suggestions from those who did were found to fall under two categories: emotional support (e.g., “exploring with the person what their fears are”), and information/advice (e.g., “to have a discussion about what to expect and maybe see pictures of what a new scar looks like”), with subcategories under each.

**Table 7.** Content analysis of responses to question: “Please describe what, if any, help you were offered whilst in care to look at your altered appearance.”

Category (n)	Sub-Category	Frequency	Example
None (46)	None offered	33	“None”
	Not applicable	2	“N/A”
	Didn’t require any	11	“None was needed…” “Did not require any help.”
Medical (11)	Instructions for aftercare	6	“I was told not to wash the area or use soap on it for several weeks…”
	Medical treatment	5	“I was offered medical treatment and regular dressings”
Emotional (7)	Support from friends and family	4	“…only support from family and friends”
	Reassurance from medical professional	3	“…the nurses were of course very kind and reassuring”

**Table 8.** Content analysis of responses to question: “Could anything have been done differently by medical professionals/nurses that may have improved your experience of seeing your altered appearance for the first time?”

Category (n)	Sub-Category	Frequency	Example
None (32)		32	“No they did what they could…”
Not sure (1)		1	“Not sure”
Do not remember (1)		1	“Don’t remember”
Emotional Support (13)	Preparation	6	“…preparing people better…” “Actually telling me what to prepare for…”
	Ask about patient's worries/fears	2	“…exploring with the person what their fears are.”
	More empathy	1	“It would’ve been nice to have a bit more empathy from the staff and nurses…”
	More care	3	“Extra care and support”
	Honesty	1	“I guess honesty and having the right people there…”
Information/Advice (12)	Expectations	6	“…to have a discussion about what to expect and maybe see pictures of what a new scar looks like…” “…heads up of the expected healing schedule…”
	Aftercare instructions	4	“…hospital discharge leaflet about expectations and aftercare would have been nice.”
	Where to seek support	1	“…how to seek support which would have been helpful to have”
	Counselling services	1	“…offering some counselling resources for body image”

Both quantitative and qualitative findings suggest approximately half of the sample did not feel any further improvements could have been made to the support they were given around this experience. However, those who did feel that support could have been improved had clear ideas of how this could be implemented.

### Demographic differences

No significant differences were found between burn injuries and surgical scarring in terms of distress (*U* = 324.00, *z* = −0.02, *p* = .986), preparedness (*U* = 282.50, *z* = −0.73, *p* = .467), and support (*U* = 307.50, *z* = −0.21, *p* = .765). Furthermore, no significant differences were found between males and females with regards to distress (*U* = 263.50, *z* = −0.96, *p* = .338), preparedness (*U* = 262.50, *z* = −0.98, *p* = .329), and support (*U* = 247.50, *z* = −1.14, *p* = .217). Similarly, no significant differences were found between age groups for distress (X^2^ (3) = 4.678, *p* = .197), preparedness (X^2^ (3) = 3.107, *p* = .375), or support (X^2^ (3) = 2.624, *p* = .453).

## Discussion

The aim of this study was to investigate participants’ initial experiences of seeing their altered appearance for the first time after surgical scarring, burn injury or amputation. This research specifically explored distress, preparedness, and perceived support surrounding participants’ initial experiences, as well as any differences according to demographics or the type of visible difference acquired. The findings highlight the range of emotions experienced at this timepoint and demonstrate the importance of perceived social support in reducing distress. Most participants felt that this was an important element of their healthcare pathway, but experienced initial worry or concern at seeing their appearance. Whilst most participants felt they received sufficient support and found the experience either as distressing or less distressing than expected, a quarter of participants suggested they would have liked more support around this time point. Moderate levels of distress were found to be associated with this experience, with 36 participants listing ‘distress’ as a reaction to seeing their appearance for the first time, and those reporting higher levels of distress found to be more likely to have less support and feel less prepared. Whilst these findings are correlational, and thus we cannot assume causation, they offer some insight into how distress could be reduced in the future. Interestingly, no differences were found between gender, age groups or the different appearance altering conditions.

These results echo previous findings exploring women's initial experiences after having breast reconstruction surgery,^7,8^ as well as exploring the experiences of individuals with facial burns.^5^ Similarly to these studies, participants expressed a range of reactions to their altered appearance, including positive interpretations (with participants simply thankful for being alive), and more negative emotions, such as shock and anger. As with previous research, although some participants displayed moderate levels of distress, the majority found the experience to be as distressing or less distressing than expected. This also echoes previous research suggesting that although the initial experience may cause distress, for most individuals this level of distress is expected.^5,7^ Nevertheless, the study results highlight an important group of participants who found the experience more distressing than they expected, and thus experiences varied vastly across participants. One reason for this heightened distress could be how prepared a participant felt, as there was a significant strong association between preparedness and distress, where those who felt more prepared experienced less distress. Preparedness was also identified as a key theme in previous research,^8^ where their findings suggest that those who are less prepared often experience worse outcomes. The importance of these initial experiences should not be underestimated, with existing research suggesting that short-term experiences can be predictive of longer-term outcomes.^6^ These findings should be interpreted tentatively, with limitations of this study explored below. However, taken as a whole, these findings suggest that it is important that participants are adequately prepared before seeing their appearance for the first time, that patients’ expectations around their changed appearance are discussed, and that appropriate support is provided in line with patients’ preferences.

Interestingly, no significant differences were found in distress levels according to gender or age, reflecting the existing literature.^5^ Furthermore, no differences were found in the experiences or levels of support between patients with burn injuries and patients with surgical scars. However, the sample included in this study was relatively small, with only two types of appearance change represented in this sample and unequal numbers of participants in these groups, and further research would benefit from exploring this further,

### Limitations

Whilst this study is thought to make an important contribution to a growing area of research, the findings should be interpreted within the context of the study's limitations. For example, the sample of this study was smaller than an a priori power analysis recommended, as well as being predominantly young, White and female sample. In addition, only one participant had experienced amputation and therefore the results of this study cannot be generalised to this population, with more research specifically exploring the experience of amputation needed. Future studies would benefit from exploring the experiences of a more diverse sample (particularly different ethnicities) and looking at a much larger sample of individuals. As noted in the ‘Methods’ section, this study did not recruit the target number of participants and thus the results should be interpreted tentatively. A larger sample would allow for stronger statistical conclusions, as well as a wider range of experiences. This could potentially be achieved by recruiting through NHS services where changes to appearance are anticipated and approaching all patients who come through these services to take part. This would also allow prospective research to explore their experiences in real time.

The potential for self-selection bias due to recruitment through social media and existing research participant pools should also be acknowledged, with participants perhaps more likely to take part if their experience of viewing their appearance for the first time was particularly distressing or notable. Those who did not experience any distress around this time point may have seen this research as less relevant to them and been less likely to take part. Alternatively, those who experienced significant distress may have felt unable to take part due to the difficult nature of recalling this experience. In addition, the retrospective nature of recalling initial experiences may have introduced an element of recall bias, with many of the participants in this study experiencing this event more than 18 months prior. Future research could include a more detailed exploration of the duration since the experience and the current status of scarring, as well as whether the individual is currently undergoing any treatment relating to their condition or this scarring. Future research might also explore how their perceptions have changed in the time since the event and with further chance for reflection.

Finally, future research may benefit from the addition of standardised validated measured to explore constructs such as body image or self-perception. This would be more appropriate in a prospective and longitudinal study, so that researchers might explore how such elements changed over time. A more in-depth qualitative exploration of these experiences would also allow a more nuanced understanding of many of the issues raised above, with the researchers able to follow-up on many of the points raised in this paper and explore the exact meanings behind many of the feelings expressed.

### Clinical implications and future directions

This study, combined with a growing body of research in this area, highlights the need to consider the support provided around this potentially distressing timepoint for patients and develop specific interventions to support individuals through this experience. Specifically, these results suggest that patients should be given the option to communicate with a professional about their concern, fears and worries prior to seeing their appearance altering condition for the first time, to help prepare individuals for what they might see. This could be with the healthcare professionals who are likely to be supporting them when they view their appearance for the first time (e.g., nurses, healthcare assistants, doctors) and training could be provided for these individuals to appropriately equip them for these conversations. Specific interventions could be developed to guide these conversations between participant and healthcare professional. Alternatively, those patients who identify concerns around this timepoint prior to an expected change in appearance (e.g., due to surgical scarring) may benefit from referral to counselling services, as well as those who experience distress as a result of this experience.

Future research in this area could investigate whether the anticipated visibility of the appearance altering condition to others influences initial patient experiences, and whether there are any differences in social support, preparedness, and distress in these groups. Furthermore, a larger sample involving a more equal representation between genders, age groups and appearance altering conditions would be beneficial to assess the generalisability of these findings. Whilst the mixed methods approach taken in the current study allowed some more contextual insights into participants’ experiences, a more detailed qualitative investigation of patient experiences would allow researchers to explore the nuance of this topic in more depth.

Finally, this topic of research would benefit from longitudinal research designs, which would allow researchers to explore how initial distress and preparedness impact longer-term adjustment and psychological outcomes.

## Conclusion

In conclusion, this study builds upon previous research by investigating patients’ initial experiences of a wider range of appearance altering conditions and highlights the importance of social support and preparedness in reducing negative outcomes such as distress. This has important implications for clinicians working with patients experiencing a change to their appearance, both expected and unexpected. Overall, this study has demonstrated the individuality of patients’ initial experiences and the importance of social support and preparedness, and thus care settings should be flexible in their approach to adequately support and prepare individuals before and after seeing their appearance altering condition.
